# Chimeric Antigen Receptor Signaling Domains Differentially Regulate Proliferation and Native T Cell Receptor Function in Virus-Specific T Cells

**DOI:** 10.3389/fmed.2018.00343

**Published:** 2018-12-11

**Authors:** Bilal Omer, Paul A. Castillo, Haruko Tashiro, Thomas Shum, Mai T. A. Huynh, Mara Cardenas, Miyuki Tanaka, Andrew Lewis, Tim Sauer, Robin Parihar, Natalia Lapteva, Michael Schmueck-Henneresse, Malini Mukherjee, Stephen Gottschalk, Cliona M. Rooney

**Affiliations:** ^1^Center for Cell and Gene Therapy, Baylor College of Medicine, Houston Methodist Hospital, Texas Children's Hospital, Houston, TX, United States; ^2^Department of Pediatrics, Baylor College of Medicine, Houston, TX, United States; ^3^Department of Pathology and Immunology, Baylor College of Medicine, Houston, TX, United States; ^4^Department of Molecular Virology and Microbiology, Baylor College of Medicine, Houston, TX, United States

**Keywords:** adoptive cell therapy, chimeric antigen receptor, virus specific t cells, t cell receptor, signaling domains

## Abstract

The efficacy of T cells expressing chimeric antigen receptors (CARs) for solid tumors has been limited by insufficient CAR T cell expansion and persistence. The use of virus-specific T cells (VSTs) as carriers for CARs may overcome this limitation since CAR-VSTs can be boosted by viral vaccines or oncolytic viruses. However, there is limited understanding of the optimal combination of endodomains and their influence on the native T cell receptor (TCR) in VSTs. We therefore compared the function of GD2.CARs expressing the TCR zeta chain (ζ) alone or combined with endodomains from CD28 and 4-1BB in varicella zoster virus-specific (VZV) T cells. VZVSTs expressing GD2-CARs recognized VZV-derived peptides and killed GD2-expressing tumor cells. However, after repeated stimulation through their native TCR, the expansion of GD2-CAR.CD28ζ-VZVSTs was 3.3-fold greater (*p* < 0.001) than non-transduced VZVSTs, whereas GD2-CARζ- and GD2-CAR.41BBζ inhibited VZVST expansion (*p* < 0.01). Compared to control VZVSTs, GD2-CAR.ζ VZVSTs showed a greater frequency of apoptotic (*p* < 0.01) T cells, whereas prolonged downregulation of the native αβ TCR was observed in GD2-CAR.41BBζ VZVSTs (*p* < 0.001). We confirmed that CD28ζ can best maintain TCR function by expressing GD2.CARs in Epstein-Barr virus-specific T cells and CD19-CARs in VZVSTs. In response to CAR stimulation VSTs with CD28ζ endodomains also showed the greatest expansion (6 fold > GD2-CAR.41BBζ VZVSTs (*p* < 0.001), however anti-tumor efficacy was superior in GD2-CAR.41BBζ-VZVSTs. These findings demonstrate that CAR signaling domains can enhance or diminish the function of the native TCR and indicate that only CD28ζ may preserve the function of the native TCR in tonically signaling CAR-VSTs.

## Introduction

Chimeric antigen receptors (CARs) redirected to the B-cell antigen CD19 have yielded impressive clinical results, associated with exponential CAR-T cell expansion in patients with B cell acute lymphoblastic leukemia (B-ALL) and lymphoma (1–4). However, CAR-T cells targeting solid tumors have been less effective, as they fail to expand sufficiently after infusion despite the presence of costimulatory endodomains. This failure is likely in part because solid tumors lack the costimulatory molecules expressed by B-cells and instead express inhibitory cytokines and ligands, which prevents adequate T cell expansion at the tumor site (5). Hence, the successful treatment of solid tumors might require additional strategies to preserve and enhance the *in vivo* function of CAR T cells.

One means to enhance the *in vivo* proliferation and function of CAR-T cells is via their native T cell receptor (TCR). Because most viruses potently activate both innate and TH1 polarized immunity, viral vaccines or oncolytic viruses could enhance the activity of CAR-transduced virus-specific T cells (VSTs) through their native TCRs. In a clinical trial of patients receiving VSTs transduced with a CD19.CAR possessing a CD28ζ signaling domain, we observed that patients with Epstein-Barr virus (EBV) reactivation exhibited a concomitant increase in both EBV-specific T cells and the CD19.CAR signal, suggesting virus-induced expansion of CD19.CAR-VSTs (6).

Our center demonstrated that cytomegalovirus (CMV)-specific VSTs engrafted with a CAR specific for the disialoganglioside GD2 (GD2.CAR) can be boosted with a CMV vaccine in a mouse model of neuroblastoma, leading to improved tumor control (7). A clinical trial to boost Varicella Zoster Virus (VZV)-specific T cells engrafted with GD2.CAR with a commercially available VZV vaccine based on promising preclinical results (8) is ongoing (NCT01953900).

CAR-T cells employed in most clinical studies are however activated using CD3 and CD28 antibodies (non-specifically Activated T Cells—ATCs); thus, their TCR specificity is unknown and cannot be utilized for T cell stimulation. The generally preferred costimulatory endodomain for GD2.CARs in ATCs is derived from CD137 (4-1BB) because it produces superior CAR persistence compared to the CD28 signaling domain (9), but whether this is true for VSTs that possess intrinsic long-term memory potential is unknown (10–12).

To identify a CAR that functions optimally in VSTs and preserves TCR functions, we evaluated first and second generation GD2.CARs containing costimulatory endodomains derived from 4-1BB or CD28 in T cells specific for varicella zoster virus (VZVSTs) and EBV (EBVSTs). A GD2.CAR containing both CD28 and ζ (GD2.CD28ζ) enhanced the functions of VSTs compared to GD2.CARs containing 4-1BB and ζ (GD2.41BBζ) or ζ alone (GD2.ζ) exhibited reduced VST proliferation and cytokine secretion in response to TCR stimulation and decreased expansion when stimulated through the CAR. Decreased proliferation of CAR-VSTs was associated with increased apoptosis in GD2.ζ modified VSTs and downregulation of native TCRs in GD2.41BBζ VSTs. Thus, our results indicate that only CARs with CD28 signaling domains are able to maintain their full TCR mediated antiviral activity.

## Materials and Methods

### Cells and Cell Lines

Blood was collected from healthy donors under a Baylor College of Medicine (BCM) Institutional Review Board approved protocol. Peripheral blood mononuclear cells (PBMCs) were separated with Lymphoprep^TM^ solution (Stemcell Technologies, Vancouver, BC, 07801). All cell lines were tested for mycoplasma prior to freezing. K562 cells, genetically engineered to express CD80, CD83, CD86, and 4-1BBL (K562cs; kind gift of Dr. Carl June, University of Pennsylvania, Philadelphia, PA) (13), was obtained from a K562cs cell master bank produced by the Good Manufacturing Practices facility of the Center for Cell and Gene Therapy, BCM, TX. K562cs cells were maintained in RPMI 1640 medium (Hyclone, Logan, UT, SH3002701) supplemented with 10% fetal bovine serum (Hyclone, 10-082-139) and 2 mM GlutaMAX™-I (Invitrogen, Carlsbad, CA, 35050-061), and was not cultured for more than 3 months after thaw. LAN-1 cells (Sigma Aldrich; 06041201-1VL) were cultured in Dulbecco's Modified Eagle's Medium (DMEM; Hyclone, SH30081.01) supplemented with 10% fetal bovine serum and 2 mM GlutaMAX™-I.

### Plasmid Construction and Retrovirus Production

**Figure 1:**
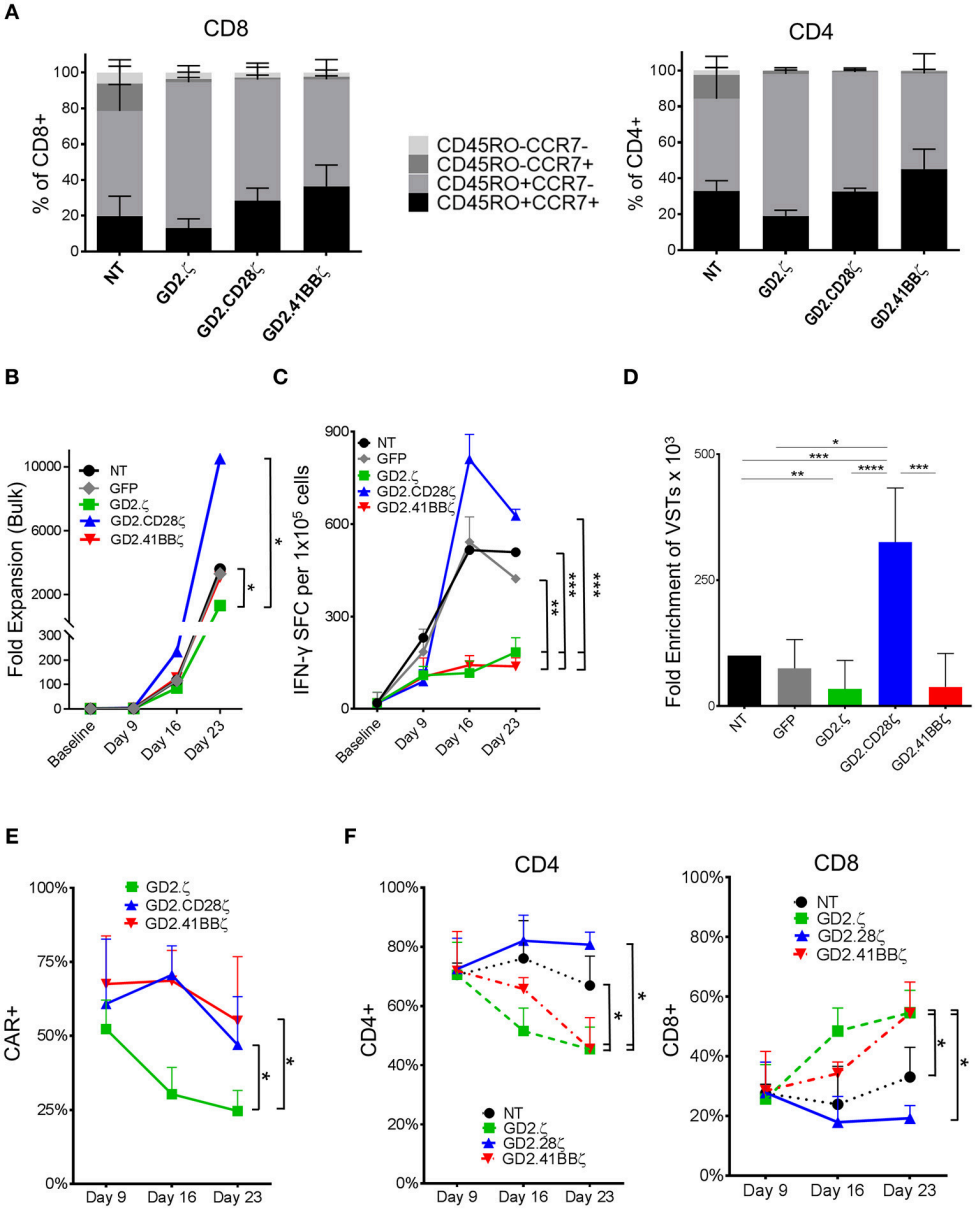
GD2.ζ and GD2.41BBζ inhibit and GD2.CD28ζ enhances the expansion of IFN-γ reactive VZVSTs after stimulation via the TCR. VZVSTs were generated as described (*n* = 7) and restimulated through the TCR on days 9 and 16 using pepmix-pulsed autologous activated T cells and irradiated K562cs cells. **(A)** CAR-transduced VZVSTs gated on the CAR and non-transduced (NT) VZVSTs were analyzed for CCR7 and CD45RO expression in CD8+ and CD4+ subsets on day 9. **(B)** The cells were counted after each TCR stimulation on days 9, 16, and 23 and the median cell numbers are shown. **(C)** Functional specificity: The frequency of T cells that produced IFN-γ in response to stimulation with VZV pepmixes was measured on the indicated culture days using ELISpot assays. **(D)** Expansion of viral antigen-specific T cells: The absolute numbers of virus-specific T cells was calculated based on the frequency of cells that secreted IFN-γ in response to viral pepmixes and the total fold expansion over 23 days. **(E)** The stability of CAR expression: The frequency of CAR-transduced cells was determined by flow cytometry on the days indicated (*n* = 7). **(F)** The proportion of CAR+ CD4+ and CD8+ cells after each TCR stimulation was determined on days 9, 16, and 23 by flow cytometry. Data for **(A–E)** are mean ± SD with *p < 0.05, **p < 0.01, ***p < 0.001, and ****p < 0.0001.

The GD2.CAR constructs (Figure 1) and the CD19.CAR constructs (Supplementary Figure S3a) have been described previously (14, 15). All GD2.CAR vectors contained a single chain variable fragment (scFv) derived from the 14g2a GD2 antibody as the antigen-recognition domain. A short IgG1 hinge connected the scFv to the CD28 transmembrane domain, and was followed by signaling endodomains from (i) the TCR ζ chain only (GD2.ζ), (ii) the CD28 endodomain fused with the ζ chain (GD2.CD28ζ), or (iii) the 4-1BB endodomain fused with the ζ chain (GD2.41BBζ). A retroviral vector expressing GFP based on the SFG backbone was used as control vector. Retroviral supernatants were produced by transient transfection of 293T cells a previously described. Briefly, 293T cells were co-transfected with each plasmid encoding the SFG retroviral vector, Peg-Pam plasmid encoding MoMLV gag-pol, and RDF plasmid encoding the RD114 envelope using FuGENE^®;^ 6 transfection reagent (Promega, Madison, WI, E2691). Retroviral supernatant was collected 48 and 72 h post-transfection, filtered (using a 0.45-μm filter), snap frozen, and stored at −80°C.

### Preparation of APCs

To prepare dendritic cells (DCs) for the first stimulation, monocytes were isolated from fresh PBMCs by CD14 selection using magnetic-activated cell sorting (MACS®) Beads (Miltenyi, Bergisch Gladbach, Germany, 130-050-201) and cultured in DC media (CellGenix, 20801-0500), 800 U/mL granulocyte-macrophage-colony-stimulating factor [GM-CSF; Fisher Scientific, PHC2011], and 1,000 U/mL IL-4 (R&D Systems, Minneapolis, MN, 204-IL) for 5 days. IL-4 and GM-CSF were replenished on day 3. On day 5, DCs were matured using a cytokine cocktail containing 10 ng/mL IL-6, 10 ng/mL IL-1β, 10 ng/mL TNF-α (R&D Systems, 206-IL, 201-LB, 210-TA, respectively), 1 mg/mL prostaglandin E2 (PGE2; Sigma, St Louis, MO, P 5640), 800 U/mL GM-CSF, and 1,000 U/mL IL-4 for 48 h.

To prepare antigen presenting cells for the second stimulation as described by Ngo et al. (16), PBMCs (1 × 10^6^ cells/ well) were stimulated on non-tissue culture 24-well plates coated with a CD3 antibody produced by the OKT3 hybridoma (ATCC #CRL 8001) and CD28 antibody (Becton Dickinson BD, Franklin Lakes, NJ, 348040) each at 1 mg/mL. ATCs were maintained in T cell medium [RPMI-1640 (Hyclone) supplemented with 45% Click's medium (Irvine Scientific, Santa Ana, CA, 9195), 2 mmol/L GlutaMAX™ TM-I (Invitrogen, Carlsbad, CA), 5% human AB Serum (Valley Biomedical, Winchester, VA)] and IL-2 (50 U/mL; NIH, Bethesda, MD), which was replenished every 3 days. Two days before antigen-specific T cell restimulation, the ATCs were reactivated on CD3/28 MAb-coated plates, peptide-pulsed, and irradiated at 30 Gray (Gy) using an RS2000 X-ray irradiator (RadSource, Suwanee, GA) immediately before being used as APCs. The K562cs cells were irradiated at 100 Gy, washed, and combined with pepmix-pulsed ATCs to stimulate effector T cells.

### Generation of VSTs

Mature DCs were pulsed with pepmixes (JPT Peptide Technologies, Berlin, Germany) comprising 15-mer amino acid peptides that overlapped by 11 amino acids and covered the entire protein sequence of the VZV antigens (ORF10, IE62, IE61, IE63, and gE) or the EBV antigens (BARF1, EBNA1, EBNA3a, EBNA3b, EBNA3c, LMP1, and LMP2). The pepmix-pulsed DCs were then co-cultured with fresh autologous PBMCs at a PBMC to DC ratio of 1:20 in T cell medium containing 10 ng/mL IL-7 and 400 U/mL IL-4 (17). On day 9 and weekly thereafter, the T cells were restimulated at a responder T cell: pepmix-pulsed autologous ATC: K562cs ratio of 1:1:3 (16).

### Transduction of VSTs and ATCs

The transduction of VSTs or ATCs was conducted 2 days after the first stimulation. The day before transduction, non-tissue culture 24-well plates were coated with 7 μg of RetroNectin® (Takara, Mountain View, CA, T100B) per 1 mL of Dulbecco's Phosphate Buffered Saline (Sigma-Aldrich, D8537) in each well and stored at 4°C overnight. The retroviral supernatant (1.5–2 mL) was added to each well and the plates were centrifuged at 2,000 × *g* for 1 h at 25°C to allow the virus to adhere to the plates. The supernatant was removed prior to the addition of the cells and the medium to eliminate inhibitory factors. VSTs (0.5 × 10^6^) were added to each well-containing T cell media supplemented with 10 ng/mL IL-7 and 400 U/mL IL-4 (18). The plates were then centrifuged at 1,000 × *g* for 5 min at room temperature and incubated at 37°C in 5% CO_2_ for an additional 7 days before functional assays were performed.

### Enzyme-Linked Immunospot Assay

Enzyme-linked immunospot (ELIspot) analysis was used to determine the frequency and function of T cells secreting IFN-γ in response to pepmixes encoding VZV or EBV antigens (18). Typically, 1 × 10^5^ T cells were plated in triplicate and stimulated with combinations of either EBV or VZV pepmixes at 0.1 mg per peptide per well. PBMCs stimulated with Staphylococcal enterotoxin B (1 μg/mL; Sigma-Aldrich, S4881) or T cells stimulated with phytohemagglutinin (5 μg/mL; Sigma-Aldrich, L8902) served as positive controls. Non-stimulated PBMCs or T cells served as negative controls. After incubation for 18 h, plates were developed for IFN-γ+ spots, dried overnight at room temperature, and sent to ZellNet Consulting (New York, NY) for quantification. The frequency of T cells specific for each antigen was expressed as specific spot-forming cells (SFCs) per input cell number. The ELISpot responses were calculated by subtracting antigen-specific SFCs from negative control SFCs.

### Flow Cytometry

Approximately 500,000 cells were washed once with FACS buffer [PBS (Sigma, P3813) containing 1% fetal bovine serum], pelleted, and antibodies were added in appropriate amounts. After incubation for 20 min at 4°C in the dark, the cells were washed with FACS buffer. The GD2.CAR was detected with the 1A7 idiotypic antibody (19) followed by PE-conjugated rat anti-mouse IgG. The cells were stained with CD25, CD3, CD4, CD8, CD45RA, CD45RO, CCR7, CD62L, TCR γ/δ, CD56, CD28, PD-1, LAG-3, and CD95 monoclonal antibodies (Becton Dickinson, Franklin Lakes, NJ) TCR α/β (Biolegend, #306718) for phenotyping, and with Pro5® CLG Pentamer (ProImmune Ltd., Oxford, UK) for EBV A^*^02:01 LMP-2 (426–434) specific T cells. To stain for apoptosis, 100,000 cells were removed from culture, resuspended in 1X binding buffer (Becton Dickinson BD) with 5 μL of annexin V (Becton Dickinson BD,) and 4 μL of 7-AAD (Becton Dickinson BD,), and incubated at room temperature for 15 min. The cells were then immediately assessed after incubation without a washing step.

### Proliferation in Response to CAR

VSTs and ATCs were generated as described. On day 11, cells were harvested for stimulation via the CAR. Cells were plated at a density of 0.5 × 10^6^ cells per well in a 24-well plate at a ratio of 1:1 with 100 Gy irradiated LAN-1 cells in the presence of 20 U/mL IL-2. The cells were counted and re-plated weekly with irradiated LAN-1 cells.

### Cytotoxicity Assay

LAN-1 target cells modified with firefly luciferase (LAN1.ffLuc) were plated in a 96-well black plate at a density of 20,000 cells/well and T cells were added at different effector to target cell ratios. After 4-h of co-culture, luciferin was added to each well and luminescence was quantified using an Infinite^®;^ M200 plate reader (Tecan, Switzerland). The number of viable LAN1.ffLuc cells in each well was calculated based on a standard curve generated from serial dilutions of the target cells. T cell cytotoxicity was calculated using the following formula: cytotoxicity % = [(cell number in the control well–cell number in the assay well) × 100]/cell number in the control well (LAN1.ffLuc cell alone).

### Immunoassays

Human IFN-γ and a human Fas ligand ELISA (R&D Systems, Minneapolis, MN, DIF50/DFL00, respectively) were used to determine the concentration of IFN-γ and soluble Fas ligand released into the culture medium by VSTs and ATCs. Cell cultured supernatants were collected after 24 h of culture as described in each experiment, centrifuged to eliminate the remaining cells, and snap frozen in three aliquots. The supernatants were thawed immediately before the assay and analyzed per the manufacturer's protocol (Tecan).

### *In vivo* Model

3 × 10^6^ Firefly-luciferase labeled LAN-1 cells were injected subcutaneously in the left flank. After 8 days 1 × 10^7^ T cells were injected intravenously and tumor growth was assessed by weekly bioluminescent imaging (Small Animal Imaging Core Facility, Texas Children's Hospital). Animal experiments were performed according to Institutional Animal Care and Use Committee approved protocols.

### Statistical Analysis

Student's *t*-test was used to test for significant differences between each set of values, based on the assumption of equal variance. The average values ± standard deviation (SD) or standard error of the mean (SEM) are provided. A *p*-value < 0.05 was considered statistically significant for all analyses.

## Results

### Differential Effects of GD2.CAR Signaling Domains on the Expansion of VZVSTs Following Stimulation Via the TCR

VZVSTs were generated by stimulating PBMCs with autologous DCs pulsed with VZV pepmixes. VZVSTs were transduced with retroviral vectors encoding the GD2.CAR constructs GD2.ζ, GD2.CD28ζ and GD2.41BBζ, which differed only in their intracellular costimulatory endodomains (Supplementary Figure S1) (17). After 9 days of culture, over 91% of cells in all groups were CD3+ T cells and over 70% expressed CD4, which is typical for VZVSTs (20). CD28 expression was decreased in GD2.CD28ζ (*p* < 0.01) and GD2.41BBζ CAR-VSTs. The GD2.CAR transgene could be detected on the surface of 52% to 75% of the GD2.CAR groups (Supplementary Figures S2a). The distribution of naïve, T central memory, T effector memory, and CD45RO negative T effector memory cells was determined by the expression of CCR7 and CD45RO (21) (Figure 1A, Supplementary Figures S2 b,c). VSTs transduced with GD2.CD28ζ and GD2.41BBζ CARs contained a trend toward higher frequencies of CD45RO+CCR7+ central memory T cells than the non-transduced (NT) and GD2.ζ-transduced cells, in both the CD4 and CD8 subsets.

VZVSTs were restimulated with VZV pepmix-pulsed APCs (16) on days 9 and 16 and their relative rate of growth is shown in Figure 1B. The frequency of VZV-reactive cells was determined using IFN-γ ELIspot assays. GD2.ζ and GD2.41BBζ VZVSTs exhibited reduced frequencies of VZV peptide-reactive T cells compared to NT or GFP-transduced control VSTs, whereas VZV specificity was maintained in GD2.CD28ζ VZVSTs (Figure 1C). The enrichment of VZV pepmix-reactive GD2.CD28ζ VZVSTs over three stimulations was greater than the enrichment apparent in NT VZVSTs (3.3-fold; *p* < 0.001), GD2.41BBζ VZVSTs (8.5-fold; *p* < 0.001), or GD2.ζ VZVSTs (9.5-fold; *p* < 0.0001; Figure 1D). The expression of the GD2.CD28ζ and GD2.41BBζ transgenes remained stable after TCR stimulation, whereas CAR expression in GD2.ζ VZVSTs decreased from a mean of 52.3% on day 9 to 24.6% on day 23 (Figure 1E; *p* < 0.05). The frequency of CD8+ T cells increased in the GD2.ζ and GD2.41BBζ VSTs between days 9 and 23 (Figure 1F), while the CD4/CD8 ratio remained relatively stable in NT and GD2.CD28ζ VZVSTs.

### CD19.CAR With CD28ζ Signaling Domain Enhances VZVST Expansion

To determine if the CD28ζ endodomain also enhanced the proliferation of VSTs when combined with a CAR targeting a different antigen, we evaluated two clinically tested CD19.CAR constructs in VZVSTs (Supplementary Figure S3a) (6, 22). As for GD2.CD28ζ CAR, VZV-reactivity in CD19.CD28ζ CAR-VZVSTs was enriched compared to NT-VZVSTs (7.8-fold) after three independent *in vitro* stimulations with VZV pepmixes (Supplementary Figures S3b,c; *p* < 0.05). If 4-1BB signaling was added, the frequency of VZVSTs decreased compared to the NT VZVST control (*p* < 0.01).

### GD2.41BBζ and GD2.ζ CARs Inhibit the Proliferation and Function of EBVSTs

**Figure 2:**
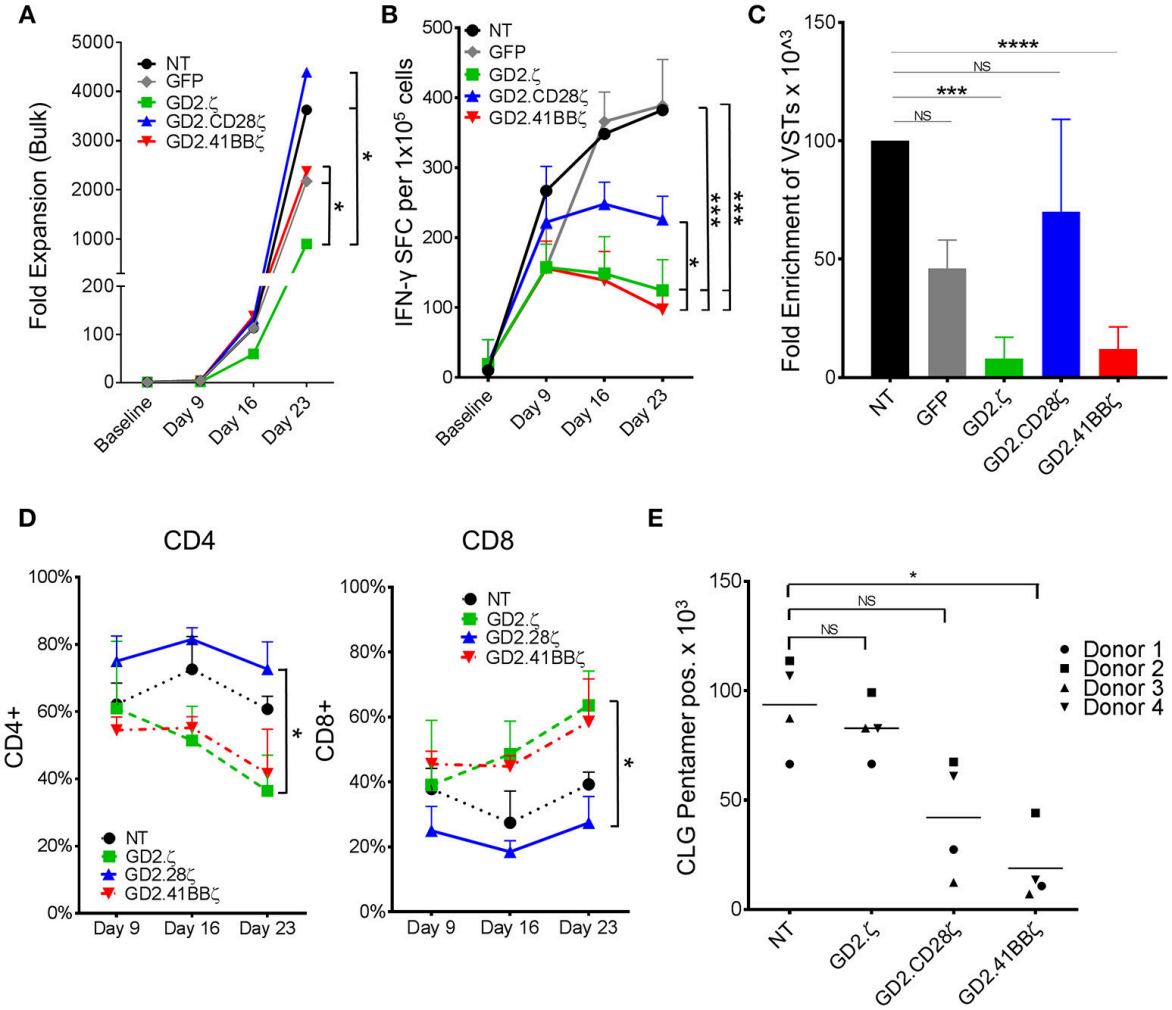
GD2.ζ and GD2.41BBζ inhibit the expansion of IFN-γ reactive EBVSTs after stimulation via the TCR. EBV-specific T cells (EBVSTs) were generated as described (*n* = 5) and restimulated through the TCR on days 9 and 16 using pepmix-pulsed autologous activated T cells and irradiated K562cs cells. **(A)** Cells were counted after each TCR stimulation on days 9, 16, and 23 and the median cell numbers are shown. **(B)** Functional specificity: The frequency of T cells producing IFN-γ in response to stimulation with viral pepmixes was measured on the indicated culture days using ELIspot assays. **(C)** Enrichment of viral antigen-specific T cells: The absolute number of virus-specific T cells was calculated based on the frequency of cells that secreted IFN-γ in response to viral pepmixes and the total fold expansion. **(D)** The proportion of CAR+ CD4+ and CD8+ cells (except NT) after each TCR stimulation was determined on days 9, 16, and 23 by flow cytometry. **(E)** NT and CAR.VSTs specific for the EBV epitope CLG (LMP2) were generated by stimulation with CLG peptides and CLG-specific cells were identified on day 9 by pentamer staining as well as antibody staining for CD8 (gated on CAR+, except NT). The absolute number of CLG-specific cells was calculated by multiplying the proliferation and the percent of pentamer+ cells (*n* = 4). Data for **(A–D)** are mean ± SD [except **(C)** = SEM] with *p < 0.05, ***p < 0.001, and ****p < 0.0001.

To determine if the inhibitory effects of GD2.41BBζ and GD2.ζ on VZVSTs applied to T cells specific for other viruses, we generated EBVSTs using pepmixes that spanned seven EBV latency-associated antigens. We evaluated the proliferation and viral antigen reactivity of EBVSTs after 3 stimulations via the TCR using antigen presenting cells (APCs) pulsed with EBV pepmixes (Figures 2A,B). As for VZVSTs, the expression of GD2.41BBζ and GD2.ζ inhibited the expansion of IFN-γ reactive EBVSTs compared to NT- and GFP-EBVSTs (Figure 2C; p < 0.05). The enrichment of GD2.CD28ζ-EBVSTs was similar to control GFP transduced and NT-EBVSTs. Similar to our findings in VZVSTs, we observed a decrease in the frequency of CD4+ T cells in GD2.ζ and GD2.41BBζ-modified EBVSTs compared to NT and GD2.CD28ζ T cells (Figure 2D).

### GD2.41BBζ CAR Decreases Antigen Specificity of EBVSTs

The reduced frequency of viral antigen reactive T cells within ζ only and 41BBζ containing GD2.CARs might reflect T cell dysfunction rather than loss of antigen-specific T cells within the bulk T cell population. Pentamer staining provides a function-independent means to measure antigen-specific T cells. By co-staining for the CAR and a human leukocyte antigen (HLA)-A2-pentamer presenting the EBV-latent membrane protein 2 (LMP2) epitope CLGGLLTMV (CLG) in four donors with CLG specific T cells, we determined that the number of antigen specific T cells by pentamer staining decreased by 80% in GD2.41BBζ compared to NT EBVSTs (*p* < 0.05; Figure 2E). A lesser, non-significant decrease was observed in GD2.28ζ EBVSTs (55%) compared to NT EBVSTs, while no decrease was seen in GD2.ζ EBVSTs, suggesting that the mechanisms underlying the loss of virus specificity were different in GD2.ζ and GD2.41BBζ VSTs: Hence while GD2.41BBζ VSTs downregulated their TCR or failed to expand, GD2.ζ EBVSTs became functionally anergic. Indeed, HLA pentamer-positive CLG-specific cells showed decreased expression of IFN-γ compared to NT EBVSTs (*p* < 0.05, data not shown).

### GD2.ζ and GD2.41BBζ Induce Apoptosis in VZVSTs Following TCR Stimulation

**Figure 3:**
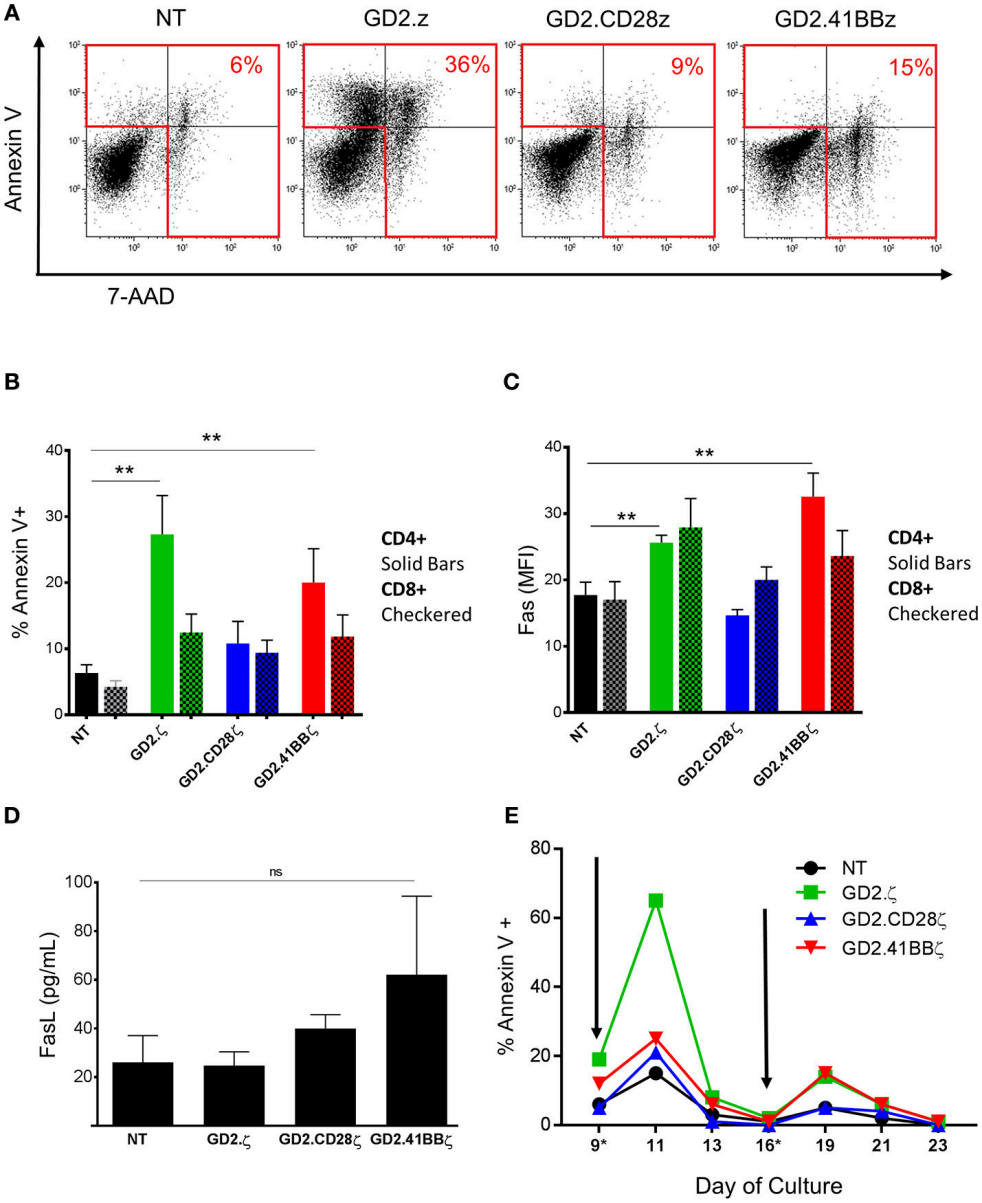
Increased apoptosis in GD2.ζ CAR modified VSTs peaks after TCR stimulation. **(A)** The viability of VSTs and ATCs was determined on day 9 using annexin V staining and 7-aminoactinomycin D (7-AAD). Cells were analyzed by flow cytometry with gating on CD3+/CAR+ lymphocytes. **(A)** Annexin V (y-axis) and 7-AAD staining in VSTs from one of four representative donors is shown. **(B)** The average proportion of annexin V+ cells in the CD4 and CD8 T cell subsets from the four donors is shown (mean ± SD). **(C)** VSTs (gated on CAR+ except for NT) were analyzed for Fas expression (CD95) on day 9 and the mean fluorescence intensity in the CD4+ and CD8+ subsets was determined (*n* = 4). **(D)** VSTs were generated as described and the supernatant was collected on day 9. The Fas ligand concentration was then determined by ELISA (*n* = 4; mean ± SD). **(E)** VSTs were generated and restimulated via the TCR using VZV pepmixes on days 9 and 16 (gray arrows) as described. The proportion of annexin V+ cells (of CAR+, except for NT) at different time points before and after stimulation is depicted in one donor. **(A–E)**: **p < 0.01.

To determine if the reduction in GD2.ζ and GD2.41BBζ VZVST expansion compared to NT and GD2.CD28ζ VZVSTs was due to reduced cell divisions or increased apoptosis, we measured the percentages of dead and apoptotic cells on day 9 of culture by 7-AAD and annexin V staining (Figure 3A). GD2.ζ and GD2.41BBζ VZVSTs exhibited higher frequencies of apoptotic cells (*p* < 0.05) compared to NT controls and GD2.CD28ζ-transduced VSTs (27.3% of CD4+ in GD2.z and 20% in GD2.41BB vs. 6.3% in NT; *p* < 0.01; Figure 3B). This increase in annexin V correlated with increased Fas expression in comparison to NT (*p* < 0.01) and GD2.CD28ζ VZVSTs (Figure 3C; *p* < 0.01). A similar trend was noted in CD8+ cells. The concentration of soluble Fas ligand was increased as measured by ELISA on day 9 was increased in GD2.41BBζ VSTs but did not achieve statistical significance (Figure 3D). When we analyzed the kinetics of apoptosis, the frequency of apoptotic VZVSTs reached a maximum 2 days after each TCR stimulation, affecting more than 60% of GD2.ζ CAR-positive cells after the second stimulation (Figure 3E).

### TCR α/β Is Downregulated in GD2.41BBζ VZVSTs and Associated With Decreased Response to Viral Antigens

**Figure 4:**
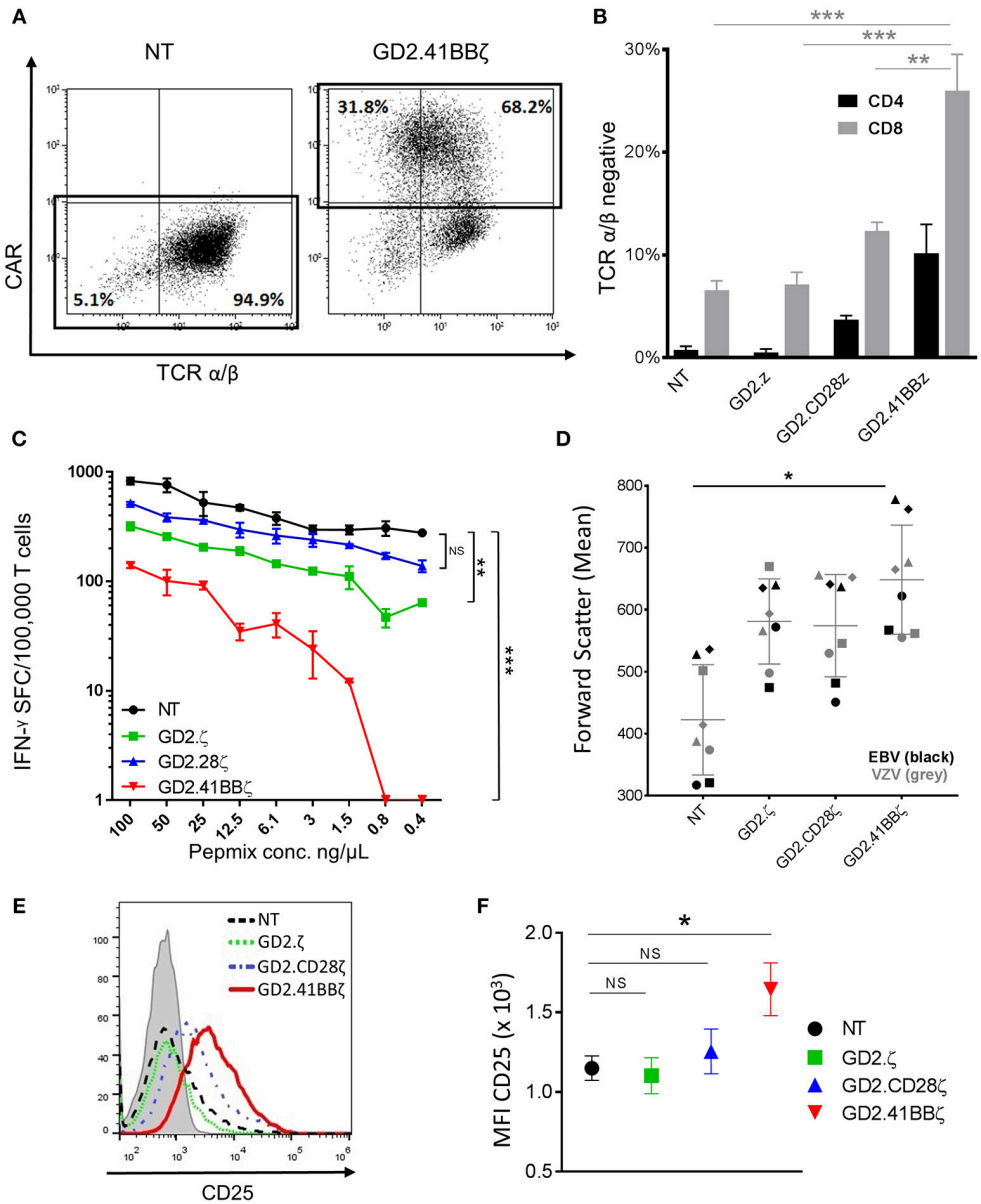
Transducing VSTs with GD2.41BBζ results in activation and downregulation of their native TCR. **(A)** VZVSTs were generated and TCR expression was analyzed by flow cytometry on day 9. The CAR expression (y-axis) and TCR α/β expression (x-axis) of NT VSTs and GD2.41BBζ VSTs from one representative donor are shown after excluding γ/δ positive cells and lymphocyte gating by forward scatter (FSC) and side scatter (SSC). **(B)** The percentage of TCR α/β negative cells was determined in CD4+ and CD8+ T cell subsets after excluding γ/δ positive cells and gating on the CAR (except NT)—(*n* = 4; mean ± SD). **(C)** VZVSTs were stimulated with limiting dilutions of VZV pepmix and the number of IFN-γ-secreting cells at each concentration as measured by ELIspot is shown for one representative donor. **(D)** The mean values of forward scatter as a marker of cell size were determined by flow cytometry in VZVSTs and EBVSTs on day 9 (*n* = 4 EBV and *n* = 4 VZV). **(E,F)** The cells were stained for the activation marker CD25 and analyzed by flow cytometry (gated on CD8+ and CAR+, except NT). A representative donor is depicted in **(E)** and the mean fluorescent intensity of four donors is shown in **(F)**. *p < 0.05, **p < 0.01 and ***p < 0.001.

To further investigate the mechanisms underlying the disparate effects of different CAR endodomains on the function of the native TCR, we evaluated TCR α/β expression using flow cytometry and observed a marked reduction in the mean fluorescent intensity of TCR α/β in GD2.41BBζ compared to NT VZVSTs (Figure 4A). A mean of 26% of CD8+ and 10.2% of CD4+ T cells transduced with GD2.41BBζ had downregulated their TCR α/β receptor (vs. 6.5% of CD8+ and 0.8% of CD4+ in NT; *p* < 0.01; Figure 4B). Further, co-staining with γ/δ, CD3, and CD56 antibodies confirmed that this loss of TCR α/β was not due to the outgrowth of TCR γδ T cells or NK cells (not shown). Downregulation of the TCR in GD2.ζ appeared similar to NT VZVSTs and slightly increased in GD2.28.ζ VZVSTs.

To determine if TCR downregulation in GD2.41BBζ VSTs affected their functional avidity, we cultured VZVSTs with limiting dilutions of VZV pepmixes and evaluated IFN-γ secretion by ELIspot. In a representative donor depicted in Figure 4C, IFN-γ secretion at lower peptide concentrations was moderately decreased by 67% (0.8 ng/μL) and 63% (0.4 ng/μL) in NT VSTs, but was lost in GD2.41BBζ VZVSTs; titration curves for GD2.CD28z VSTs were similar to NT VZVSTs. Transient downregulation of the TCR is associated with TCR activation and we therefore, evaluated cell size and CD25 expression as correlates of activation. We found that GD2.41BBζ VSTs exhibited an increase in mean cell size, as indicated by forward scatter measurement (Figure 4D; *p* < 0.05 vs. NT), and higher CD25 expression (Figures 4E,F; *p* < 0.05 vs. NT in CD8+ T cells) compared to VSTs transduced with the other CAR constructs and NT VSTs, with < 1% of cells expressing regulatory T cell markers FoxP3 and Helios in all conditions (not shown). We found a strong correlation between expression of activation markers and TCR α/β downregulation (R^2^: 0.95; *p* < 0.001).

### GD2.41BBζ Transduced VSTs Proliferate Poorly in Response to CAR Stimulation, but Maintain Anti-tumor Efficacy

**Figure 5:**
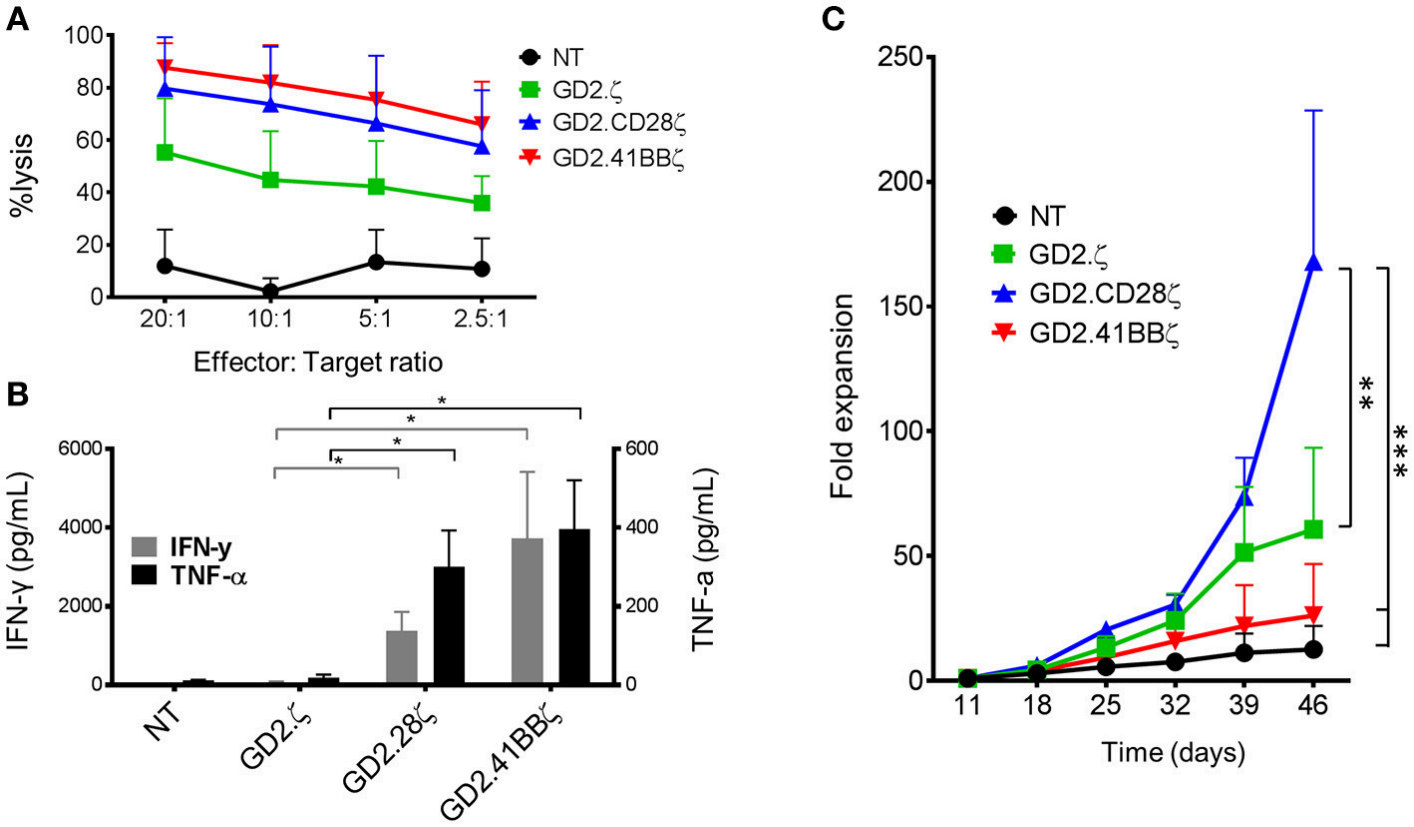
GD2.CD28ζ transduced VSTs kill target cells, secrete cytokine and show highest proliferation in response to CAR stimulation. For all experiments, CAR-VZVSTs were diluted with non-transduced (NT) cells to achieve 50% transduced cells in all CAR conditions. **(A)** Cytotoxicity: On day 9 of culture, NT and CAR-modified VSTs were added to firefly luciferase-labeled GD2-expressing LAN-1 neuroblastoma cells at the indicated ratios of effector to target cells. After a 4-h co-culture, the luminescence was quantified and T cell cytotoxicity was calculated. **(B)** Proliferation: On day 11 of culture and weekly thereafter, CAR-modified and NT VSTs were stimulated via the CAR using irradiated, GD2+ LAN-1 neuroblastoma cells at a 1:1 ratio (1:2 ratio of transduced cells to tumor cells) in the presence of low dose IL-2 (20 IU/mL). The cells were harvested and counted weekly. **(C)** Cytokine production: On day 11 of culture, VSTs were stimulated with irradiated LAN-1 neuroblastoma cells at a 1:1 ratio (1:2 ratio of transduced cells) in the absence of cytokines. After 24 h, the supernatant was collected and the concentrations of IFN-γ and TNF-α were measured by ELISA. Data are mean ± SD from for donors with *p < 0.05, **p < 0.01, and ***p < 0.001.

After examining the responses of CAR-VSTs to stimulation via the TCR, we evaluated whether different endodomains would also affect CAR-VST responses to CAR stimulation. Regardless of the endodomain(s), GD2.CAR.VZVSTs killed GD2-positive LAN-1 neuroblastoma cells at effector to target ratios ranging from 20:1 to 2.5:1 (Figure 5A). As expected, second generation GD2.CARs produced higher levels of IFN-γ and TNF-α in response to irradiated LAN-1 cells than GD2.ζ VSTs or NT controls (Figure 5B).

To compare the effects of the different costimulatory endodomains on proliferation following repeat stimulation through the CAR, NT, and GD2.CAR VZVSTs were stimulated weekly with the GD2 expressing neuroblastoma line LAN-1 (Figure 5C). GD2.CD28ζ VSTs demonstrated the greatest CAR-mediated expansion; six-fold higher than GD2.41BBζ VSTs (*p* < 0.001) and three-fold higher than GD2.ζ VSTs (*p* < 0.01) after 5 weeks of co-culture. Despite their low *in vitro* proliferation, GD2.41BBζ VSTs exhibited anti-tumor efficacy *in vivo* in a xenograft model of neuroblastoma (Supplementary Figure S4). In this model—which only provided CAR antigen and no TCR stimulation—we subcutaneously injected firefly-luciferase labeled LAN-1 cells in NSG mice, followed by intravenous injection of 1 × 10^7^ GD2.CAR VSTs. While GD2.CD28ζ VSTs delayed outgrowth of tumors, GD2.41BBζ VSTs showed superior tumor control after week 3.

To determine if the responses to CAR stimulation were different in GD2.CAR-ATCs, the “standard” platform for CARs, we generated GD2.CAR-ATCs by stimulating PBMCs on CD3 and CD28 antibody-coated plates, followed by transduction on day 2 (Supplementary Figures S5a,b) then performed identical assays for cytotoxicity, cytokine secretion and proliferation. The frequency of transduced T cells was normalized to 50% across all conditions by dilution with NT ATCs. All CAR-ATCs killed GD2+ targets at all effector to target ratios (Supplementary Figure S5c), and second generation GD2.CARs produced the highest amount of IFN-γ and TNF-α cytokines with negligible production by GD2.ζ or NT ATCs (*p* < 0.05; Supplementary Figure S5d). However, in contrast to its effect on the proliferation of VSTs, GD2.41BBζ induced the greatest proliferation of ATCs in response to GD2 stimulation (387-fold by week 4), followed by GD2.CD28ζ ATCs (277-fold), with the lowest proliferation detected in GD2.ζ ATCs (71-fold, *p* < 0.001; Supplementary Figure S5e).

Finally, we compared expression of exhaustion markers between different GD2.CAR constructs in both ATCs and VSTs (Supplementary Figure S6). PD-1 expression was increased in all NT, GD2.ζ and GD2.CD28ζ VSTs compared to ATCs transduced with the same constructs (*p* < 0.05 or < 0.01), but was decreased in GD2.41BBζ compared to GD2.CD28ζ VSTs (*p* < 0.01). LAG-3 expression was below 20% in all tested conditions, but highest in GD2.CD28ζ VSTs.

## Discussion

The use of VSTs as a platform for CAR expression might overcome some of the limitations of CAR T cell therapy for solid tumors by promoting expansion and persistence through stimulation via the native TCR using highly immunogenic viral vaccines or oncolytic viruses. Here, we observed that the CAR signaling domains profoundly influence the function of the TCR. The expression of a first generation GD2.CAR increased apoptosis in response to TCR stimulation compared to NT VSTs, and GD2.ζ VSTs failed to respond to TCR stimulation. The addition of a costimulatory endodomain from 4-1BB in GD2.41BBζ CARs mitigated apoptosis after TCR stimulation, but the overall response to viral antigens remained low, due to the downregulation or loss of virus-specific TCRs. In contrast, VSTs expressing GD2.CD28ζ maintained robust viral-antigen mediated expansion with reduced apoptosis compared to GD2ζ or GD2.41BBζ CAR-VSTs. The finding that VST function is best preserved by GD2.CARs using CD28 signaling was demonstrated for both EBVSTs and VZVSTs and for CD19.CARs, indicating that our findings are not limited by the TCR/antigen repertoire for a specific virus.

Interference with TCR function in CAR-VSTs occurred independent of CAR ligation by antigen. Long et al. previously demonstrated that CARs can signal in the absence of CAR antigen (9). The authors suggested that this tonic signaling was likely due to dimerization of the scFv and was present in all tested CARs to a varying degree; the exception were CD19 directed CARs, which showed only mild tonic signaling. The tested CD19.CARs did exhibit some tonic signaling and in common clinical settings CD19.CARs are expected to receive ongoing stimulation of the CD19 specific receptor by ligation with B cells, which are constantly produced in the bone marrow of most patients even after remission of the treated hematological malignancy is achieved. Signaling of the ζ domain of the native TCR upon stimulation with viral antigen is superimposed on the tonic or antigen triggered ζ signaling of the CAR and may in turn modulate signaling of the native TCR.

This dual ζ signaling may explain the high rates of apoptosis in first generation GD2.ζ VSTs. The combined ζ signaling from (i) TCR activation in response to viral antigen and (ii) signaling by the 1st generation GD2.CAR in VSTs may lead to a signal imbalance, where supra-physiologic ζ signaling is not accompanied by a corresponding increase in costimulatory signals like CD28 (9, 23, 24). ζ signaling from the TCR complex is known to increase sensitivity to Fas mediated apoptosis 48 to 72 h after TCR activation (25, 26), which correlates with the peak of apoptosis we observed 48 h after TCR stimulation of GD2.ζ VSTs. This effect is likely mediated by Fas/Fas Ligand interaction (27, 28), which is the primary mechanism for sensitizing memory T cells to apoptosis after TCR activation (29). Consistent with these results, we detected higher levels of Fas expression in GD2.ζ VSTs compared to NT and GD2.CD28ζ VSTs.

While ζ signaling can promote apoptosis, CD28 costimulation has been linked to protection from anergy and Fas-mediated cell death (12, 30–32). Therefore, tonic CD28 signaling complementing ζ signaling in GD2.CD28ζ CARs may account for the lower number of apoptotic cells observed in GD2.CD28ζ VSTs compared to GD2.ζ VSTs. By contrast 41BB costimulation provided only weak protection from apoptosis in VSTs. We observed that GD2.41BBζ modification resulted in increased activation as indicated by cell size and CD25 expression compared to NT, GD2.ζ and GD2.CD28ζ VSTs. This overall increase in activation status may render GD2.41BBζ VSTs more susceptible to apoptosis (28).

Downregulation of the native TCR was most pronounced in GD2.41BBζ VSTs and led to diminished IFN-ɤ secretion in response to stimulation of the TCR with viral antigens. Transient downregulation of the TCR is a physiologic negative feedback mechanism to prevent uncontrolled T cell expansion (33) and can occur in both CD4 and CD8 subsets (34). The kinetics of downregulation is regulated by the TCR ζ chain and is dependent on the strength of the antigenic stimulus, the presence of costimulation and the continued presence of antigen (35). We hypothesize that the increased responsiveness of CD8+ cells to 4-1BB signaling (36, 37) results in greater activation and subsequently a higher degree of TCR downregulation in the CD8+ subset. Although TCR downregulation can dramatically decrease the antigen-specific release of IFN-γ, the intrinsic capability to secrete IFN-γ and other cytokines is retained and these T cells can otherwise remain highly functional (33). In the context of a bispecific T cell product that responds to both CAR and TCR stimulation, 41BBζ -mediated downregulation of the native TCR would however diminish a key component of the bispecific CAR-VST platform while CD28ζ preserves dual-targeting capacity.

GD2.41BBζ VSTs proliferated poorly in response to stimulation with GD2+ tumor cells—even less than 1st generation CAR-VSTs—while the proliferation of GD2.CD28ζ was 6-fold higher. Despite the reduced proliferation GD2.41BBζ VSTs exhibited increased anti-tumor control compared to GD2.CD28ζ VSTs, possibly related to a higher proportion of central memory cells and a lower expression of exhaustion markers in GD2.41BBζ VSTs.

A potential explanation for the differential requirements for costimulatory signaling between VSTs and ATCs is the initial, non-physiologic activation of CAR-ATCs using CD3 and CD28 antibodies. This stimulation does not provide third tier costimulation with TNF-like signals such as 4-1BB. Zhao et al. demonstrated how spatial and temporal variation of signaling through ζ, 4-1BB and CD28 differentially affects CAR function in ATCs (38) and showed that ostensibly minor variations in the relative dose and timing of CD28 and 4-1BB signaling lead to distinct functional outcomes (38). Hence T cells activated with CD3 and CD28 antibodies may respond to CAR stimulation differently from T cells stimulated with APCs that express a range of costimulatory molecules and cytokines. The different signaling requirements of ATCs and VSTs could also result from differences in the physiology of naïve and memory T cells. Theoretically CD3 and CD28 should activate all T cell subsets, so that all subsets should be represented in CAR-modified ATCs. However, in a separate study we found that 70% or more of T cells activated with CD3 and CD28 derive from the CD45RA+ CD95- naïve T cell subset, even though these naïve cells converted to a CD45RO+ phenotype in *ex vivo* culture (39). By contrast, VSTs derive primarily if not exclusively from the CD45RO subset, which have different requirements for costimulation.

Other studies have evaluated VSTs as a platform for CARs (6, 7, 10, 40–47) and have shown that dual-specific T cells can be generated, but to our knowledge, this work is the first report that directly compares CARs with CD28 or 4-1BB in the VST setting. While we tested the CAR-VSTs in a xenograft mouse model of neuroblastoma, this model is limited in that only CAR antigen is present and antiviral stimulation is not provided. Of the three tested GD2-CAR vectors only GD2.CD28ζ VSTs maintained TCR function *in vitro*, and we therefore do not expect the other two constructs to demonstrate bispecific responses *in vivo*. The main advantage of the bispecific CAR-VST platform—the massive expansion potential of CAR bearing T cells in response to TCR antigen—is inadequately tested in mice that cannot be infected with VZV or vaccinated with EBV. Most studies of CAR-VSTs used CARs with CD28 costimulatory endodomains (6, 7, 10, 40–42), and did not report detrimental effects on TCR function, which is consistent with our observation.

In summary, our results demonstrate that CARs can enhance or diminish the function of the native TCRs in VSTs, and that only CARs with CD28ζ signaling domains appear to fully maintain their TCR function. Three *ex vivo* stimulations of GD2.CD28ζ CAR VSTs through the TCR resulted in an almost 400,000 fold enrichment of antigen-specific T cells in just 3 weeks, indicating that vigorous T cell expansion can be tapped through more physiologic activation of CAR-VSTs. Thus, CAR-VSTs have the potential to improve current T cell therapy approaches for a broad range of malignancies.

## Ethics Statement

Blood from healthy donors was collected in accordance with the recommendation of the Protocol Review Committee at Baylor College of Medicine. All subjects gave written informed consent in accordance with the Declaration of Helsinki. The protocol was approved by the Institutional Review Board at Baylor College of Medicine.

## Author Contributions

CR and BO: conception and design; BO, PC, HT, ThS, MC, MH, MT, AL, TiS, RP, NL, MS-H, MM, SG, and CR: experimental design; BO, PC, HT, ThS, MC, MH, AL, and MS-H: conduction of laboratory experiments; BO, PC, HT, TSH, MC, MH, MT, AL, ThS, RP, NL, MS-H, MM, SG, and CR: analysis and interpretation of data; BO, CR, and SG: writing of the manuscript; BO, PC, HT, ThS, MC, MH, MT, AL, TiS, RP, NL, MS-H, MM, SG, and CR: review and revision of the manuscript.

### Conflict of Interest Statement

The Center for Cell and Gene Therapy has a research collaboration with Cell Medica and Tessa Therapeutics. CR and SG have patent applications in the field of immunotherapy for cancer and gene therapy for cancer. The remaining authors declare that the research was conducted in the absence of any commercial or financial relationships that could be construed as a potential conflict of interest.
