# Supplementary material for: Chimeric Antigen Receptor Signaling Domains Differentially Regulate Proliferation and Native T Cell Receptor Function in Virus-Specific T Cells
Source: Front Med (Lausanne). 2018 Dec 11;5:343. doi: 10.3389/fmed.2018.00343 (PMC6297364; doi:10.3389/fmed.2018.00343)
Supplement: Supplementary file 1 [file Data_Sheet_1.docx]

Supplementary Material

Chimeric antigen receptor signaling domains differentially regulate proliferation and native T cell receptor function in virus-specific T cells

***Bilal Omer^1,2^, Paul A Castillo^1^, Haruko Tashiro^1^, Thomas Shum^1^, Mara Cardenas^1^, Mai T.A. Huynh^1^, Miyuki Tanaka^1^, Andrew Lewis^1^, Tim Sauer^1^, Robin Parihar^1,2^, Natalia Lapteva^1^, Michael Schmueck-Henneresse^1^, Malini Mukherjee^1^, Stephen Gottschalk^1,2,3^, Cliona M. Rooney^1,2,3,4^**

*** Correspondence:** [baomer@txch.org](mailto:baomer@txch.org)

# Supplementary Figures


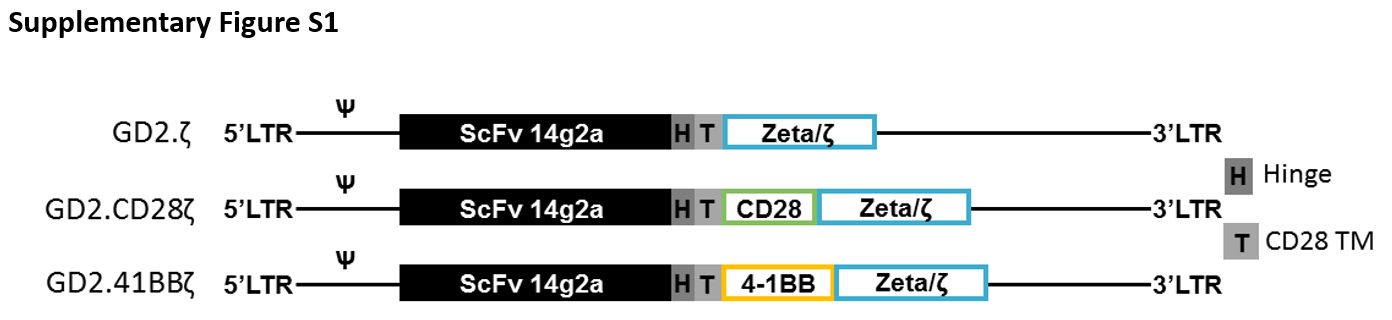


**Supplementary Figure S1:** **GD2.CAR Vector Maps.** **(a)** Schematic representation of the GD2.CAR retroviral constructs used (T=transmembrane domain, H=hinge, and LTR=long tandem repeat).

*
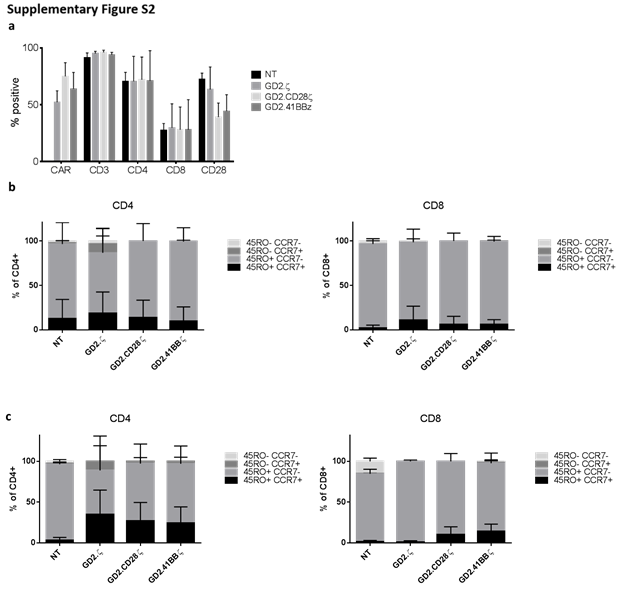
*

**Supplementary Figure S2: Phenotype of VZVSTs. (a)** Non-transduced (NT) and CAR-transduced VZV-specific VSTs (VZVSTs) were analyzed for CAR expression and the expression of the T cell markers CD3, CD4, and CD8 by flow cytometry on day 9. Data are mean ± SD from five donors**. (b-c)** NT and CAR.VSTs were gated on CAR+ (except NT gated on CD3+) and then analyzed for CCR7 and CD45RO expression in CD8+ and CD4+ T cell subsets (n=3). **(b)** Data for CD4+ and CD8+ T cells on day 16. **(c)** Data for CD4+ and CD8+ T cells on day 23.

*
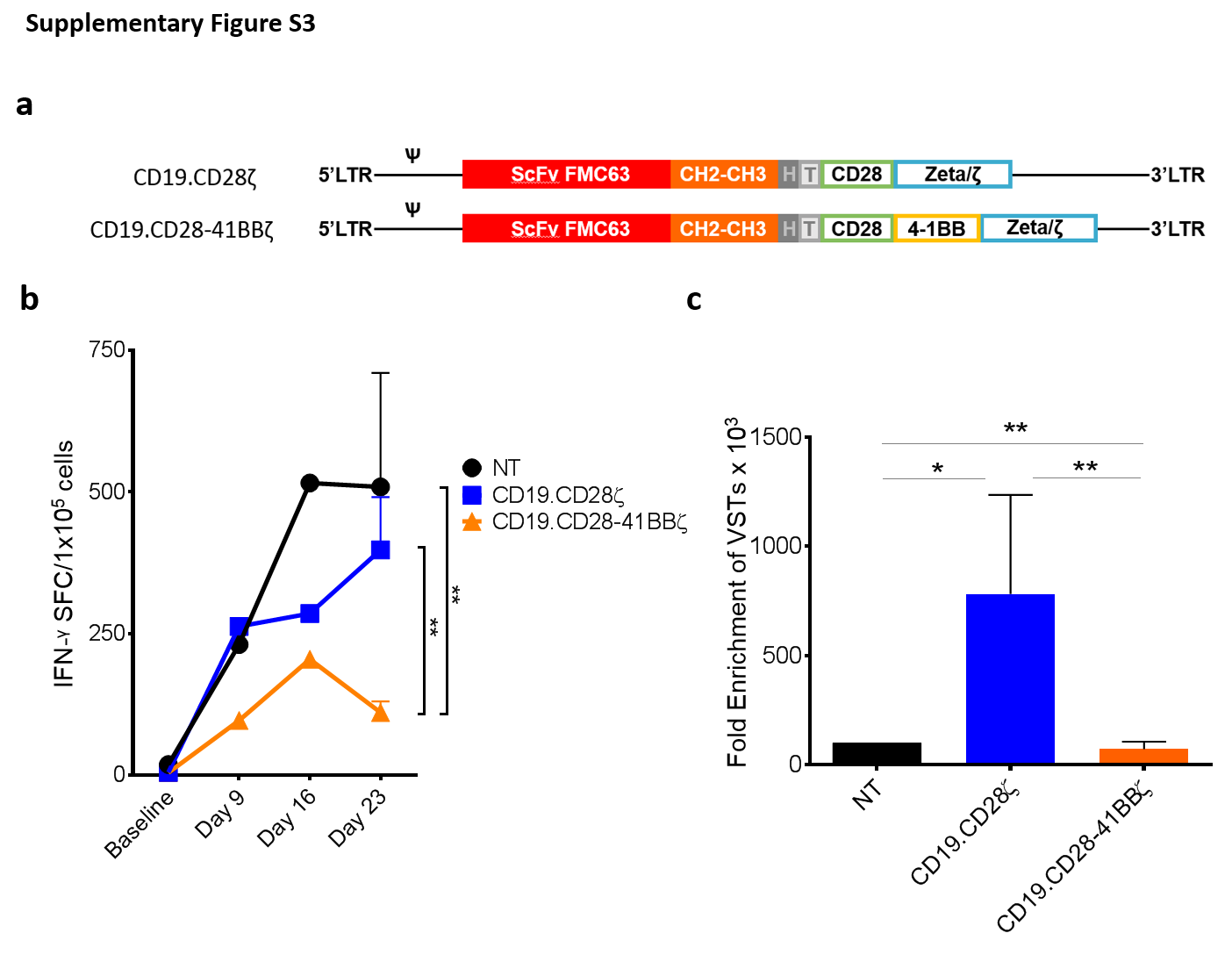
*

**Supplementary Figure S3: Effect of CD19.CAR transduction on TCR function in VSTs. (a)** Schematic representation of the retroviral CD19.CAR vectors tested (TM=transmembrane domain, H=hinge, and LTR=long tandem repeat region). **(b)** CAR.VSTs and control VZVSTs were stimulated three times through the TCR with pepmixes using our previously described protocol. The cells were counted and analyzed by ELIspot at baseline and on days 9, 16, and 23 (n=4). **(c)** The absolute numbers of virus-specific T cells were calculated based on the frequency of cells that secreted IFN-γ in response to viral pepmixes and the total fold expansion (n=4). Data for **(b,c)** are mean ± SD with *=p<0.05 and **=p<0.01.


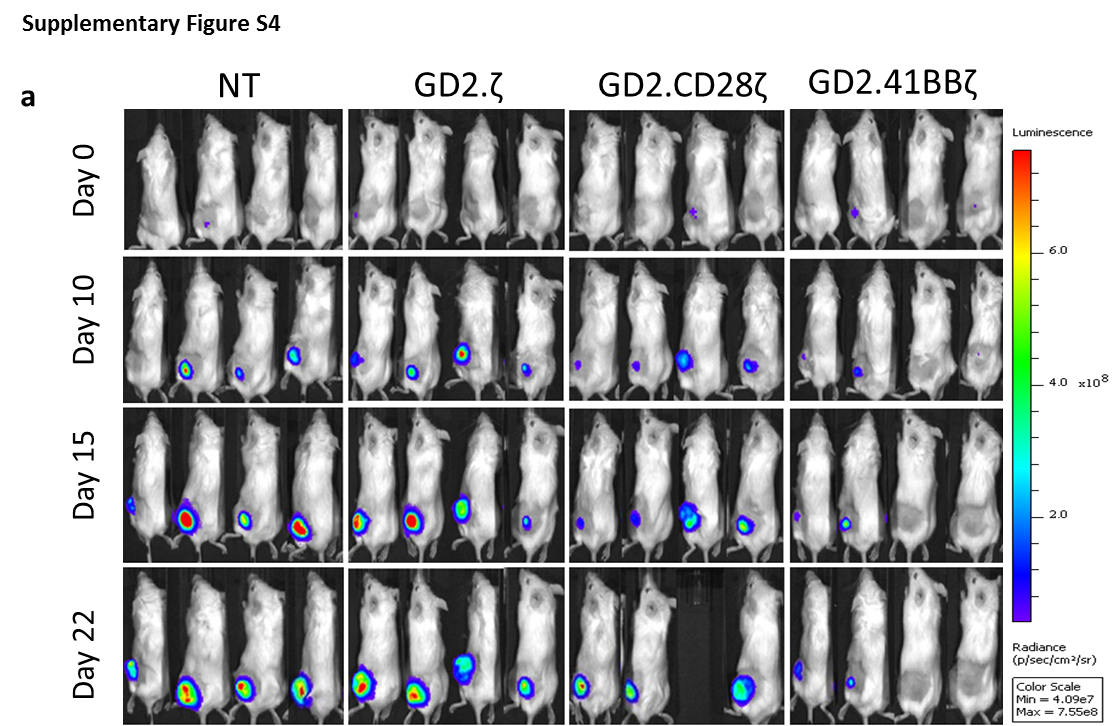


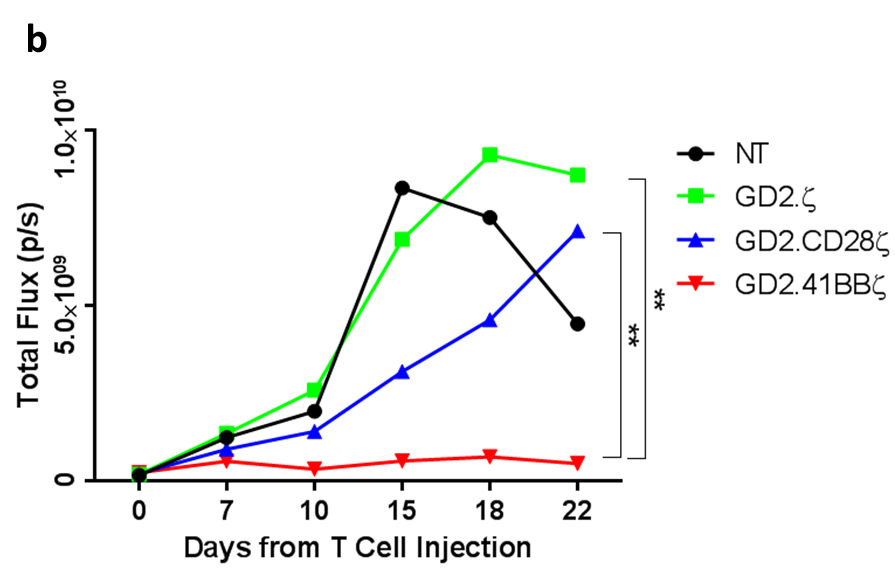


**Supplementary Figure S4: 2^nd^ Generation GD2.CAR VSTs delay tumor growth *in vivo*.** NSG mice were subcutaneously injected with 3x10^6^ firefly-luciferase modified LAN-1 cells on day -8. On day 0 mice were injected intravenously with 1x10^7^ VSTs transduced with GD2.ζ, GD.CD28ζ, or GD2.41BBζ. Non-transduced VSTs were used as a control. Tumor growth was measured biweekly with bioluminescent imaging (BLI). **(a)** BLI images depicting tumor signal over time. **(b)** Average radiance for each treatment group over time. Delayed tumor growth was observed in mice treated with GD2.41BBζ VSTs. **p<0.01.


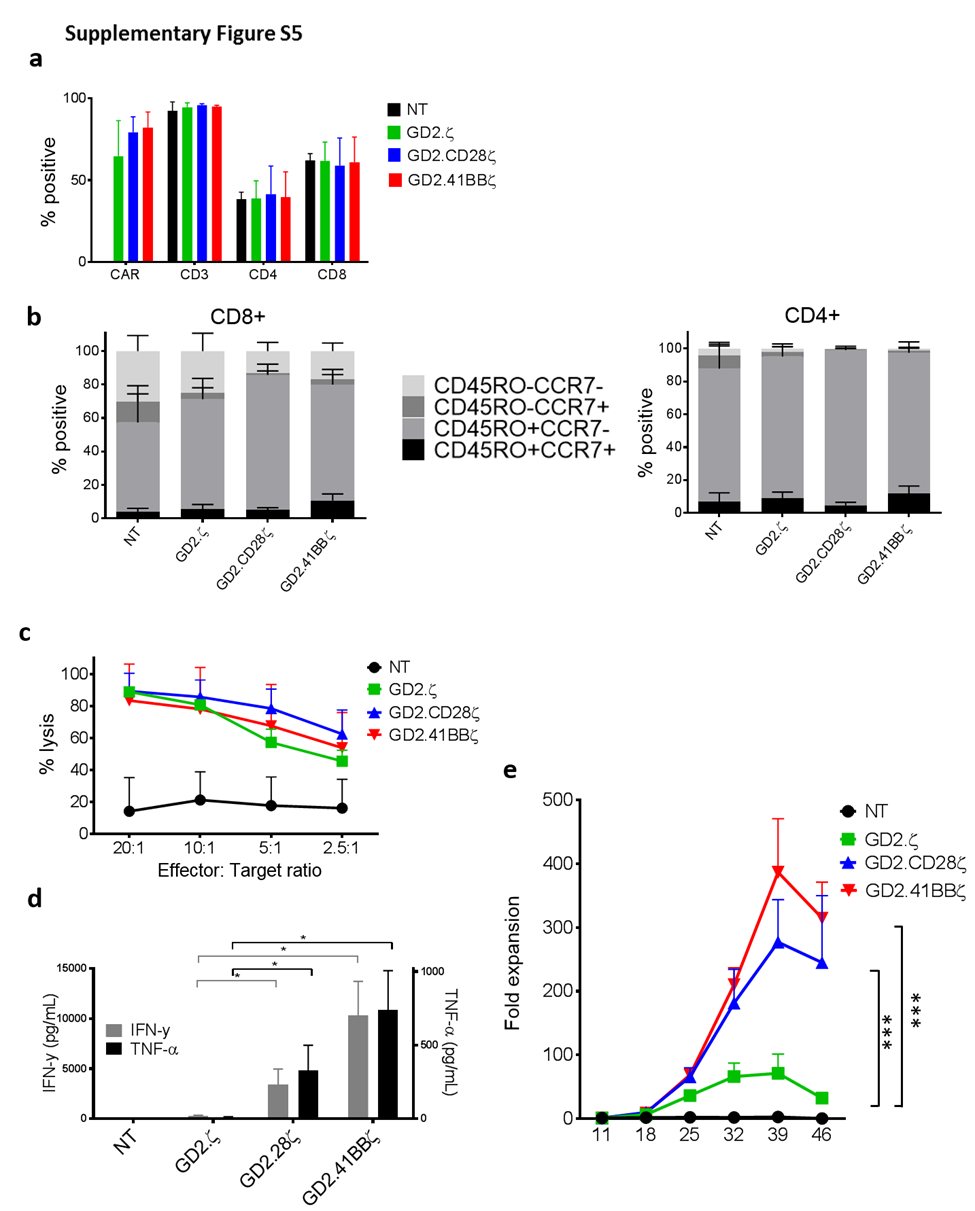


**Supplementary Figure S5: Phenotype and function of ATCs transduced with GD2.CAR vectors.** CAR.ATCs were generated by the stimulation of PBMCs on OKT3/CD28 coated plates and subsequent transduction with GD2.CAR vectors on day 2. For all experiments, CAR.ATCs were diluted with non-transduced (NT) cells to achieve 50% transduced cells in all CAR conditions. **(a)** NT and CAR-transduced cells were analyzed for CAR expression and the expression of the T cell markers CD3, CD4, and CD8 by flow cytometry on day 9. Data are mean ± SD from five donors. **(b)** NT and CAR.ATCs were gated on CAR+ (except NT) and then analyzed for CCR7 and CD45RO expression in CD8+ and CD4+ T cell subsets (n=5). **(c)** Cytotoxicity: On day 9 of culture, NT and CAR-modified ATCs were added to firefly luciferase-labeled GD2-expressing LAN-1 neuroblastoma cells at the indicated ratios of effector to target cells. After 4-hour co-culture, luminescence was quantified and T cell cytotoxicity was calculated. **(d)** Cytokine production: On day 11 of culture, the ATCs were stimulated with irradiated LAN-1 neuroblastoma cells at a 1:1 ratio (1:2 ratio of transduced cells) in the absence of cytokines. After 24 hours, supernatant was collected and the concentrations of IFN-γ and TNF-α were measured by ELISA. **(e)** Proliferation: On day 11 of culture and weekly thereafter, CAR-modified and NT ATCs were stimulated via the CAR using irradiated GD2+ LAN-1 neuroblastoma cells at a 1:1 ratio (1:2 ratio of transduced cells to tumor cells) in the presence of low dose IL-2 (20 IU/mL). The cells were harvested and counted weekly. Data for (**c-e**) are mean ± SD from four donors with *=p<0.05, **=p<0.01, and ***=p<0.001.


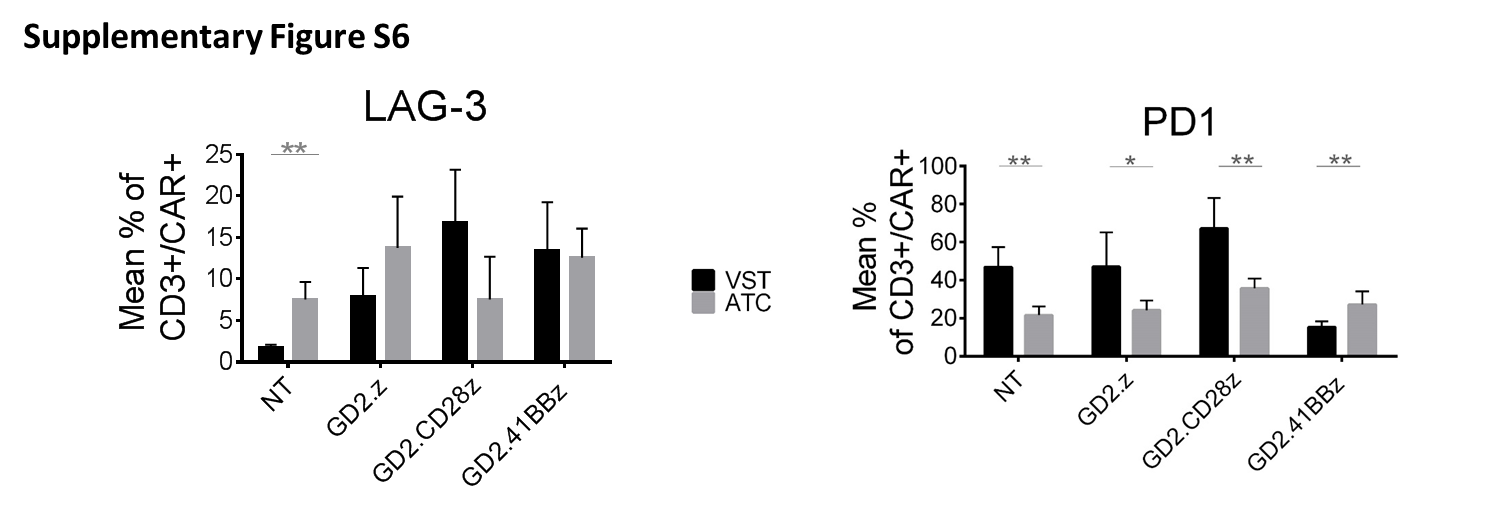


**Supplementary Figure S6: Exhaustion marker expression in VSTs and ATCs.** Non-transduced (NT) and CAR-transduced VZV-specific VSTs (VZVSTs) were analyzed for CAR expression and the expression of exhaustion markers LAG-3 and PD1 by flow cytometry on day 9. Data are mean ± SD from five donors. *=p<0.05, **=p<0.01.
